# Description of *Gattollius* gen. nov. (Ephemeroptera, Baetidae), documenting a new morphological type of accessory gill

**DOI:** 10.1093/jisesa/ieag048

**Published:** 2026-06-19

**Authors:** Chanaporn Suttinun, Boonsatien Boonsoong

**Affiliations:** Faculty of Veterinary Medicine, Chiang Mai University, Chiang Mai, Thailand; Research Center of Producing and Development of Products and Innovations for Animal Health and Production, Chiang Mai University, Chiang Mai, Thailand; Animal Systematics and Ecology Speciality Research Unit (ASESRU), Department of Zoology, Faculty of Science, Kasetsart University, Bangkok, Thailand; Biodiversity Center Kasetsart University (BDCKU), Bangkok, Thailand

**Keywords:** Oriental region, Thailand, mayfly, new genus, Acentrellini

## Abstract

A new genus, *Gattollius* Suttinun & Boonsoong gen. nov. (Ephemeroptera: Baetidae), is described from northern Thailand, together with a new species, *Gattollius thailandicus* Suttinun & Boonsoong sp. nov., based on all life stages. The nymph is unique within Baetidae in possessing accessory gills in 3 positions: a single gill on each side of the mentum, a single gill on each side between the coxa and prosternum, and a single gill at the inner proximal margin of the femur on all legs bearing a femoral patch. The adult is also distinctive in retaining accessory gills, including coxal gills in both subimago and imago, and femoral gills on each leg of the subimago. Persistence of accessory gills into the adult stage represents the first such record in Baetidae. This study further documents new type of femoral accessory gills in mayflies. These structures are not only inferred to function in respiration but also bearing femoral patch, and we propose the term “femoral villopore gills” for this specialized organ. The phylogenetic position of the genus is established, supporting its placement within the tribe Acentrellini, based on diagnostic characters observed across all life stages and may be closely related to the monotypic *Asiobaetodes* Gattolliat, 2012 from Borneo and the species *Acentrella fimbriata* Tungpairojwong, Phlai-ngam & Jacobus 2022 from Thailand.

## Introduction

The family Baetidae is the most diverse mayfly family, representing approximately one-fourth of all mayfly species worldwide. It is distributed worldwide, except for Antarctica and New Zealand, with approximately 1,100 species belonging to 110 genera. The number of genera and species of the family Baetidae reported in Southeast Asia is rising in the last decade due to increasing research interest in this region considered to have a high biodiversity (Gattolliat and Nieto 2009, Kaltenbach et al. 2020, 2021, 2022b, 2023a, 2023b, 2025, Kluge and Suttinun 2020, Suttinun et al. 2020, 2021, 2022, Kluge 2022a, Phlai-Ngam et al. 2022a, 2022b, Tungpairojwong et al. 2022). Besides being one of the most diverse families of mayflies, Baetidae comprises some monotypic genera. When we focus on the Oriental realm, there are 4 genera remaining as monotypic. The oldest monotypic genus, *Jubabaetis*  Müller-Liebenau, 1980, was described in 1980 from Philippines and is still monospecific, containing only *Jubabaetis pescadori*  Müller-Liebenau, 1980. This genus bears a unique character in the prolonged front margin of the head (Müller-Liebenau 1980). The latest monotypic genus, *Cymbalcloeon*  Suttinun, Gattolliat, & Boonsoong, 2020 from Thailand, is proposed as a unique genus due to the strange shape of its abdominal gills that are orientated ventrally and reduced to 3 in a species *Cymbalcloeon sartorii*  Suttinun, Gattolliat, & Boonsoong, 2020 (Suttinun et al. 2020). Two other monotypic genera are *Acerobiella*  Gattolliat, 2012 and *Asiobaetodes*  Gattolliat, 2012 from Borneo, Indonesia. *Acerobiella alinae*  Gattolliat, 2012 constitutes a unique character among other Baetidae by its dorsal armature, especially the spike tubercles turned upwards on tergum II. *Asiobaetodes eloi*  Gattolliat, 2012 served as a monospecific because it bears 5 pairs of ventrally orientated gills and tubular accessory gills at the bases of mouthparts and forelegs (Gattolliat 2012).

Accessory gills are rare in Ephemeroptera. An overview and phylogenetic discussions are given by Štys and Soldán 1980, Staniczek 2010, Zhou 2010, and Kaltenbach et al. 2022b. In Baetidae, the genera with accessory gills are from the Nearctic, Neotropical, Afrotropical and Oriental regions. Only 3 genera of the Oriental region were mentioned (Gattolliat 2012, Vasanth et al. 2020, Kaltenbach et al. 2022b, Phlai-Ngam et al. 2024). All of them were observed only in the larval stage and gills are present in 3 positions: coxal gills, maxillary gills, and labial gills. Coxal gills on 2 or 3 pairs of legs were observed in the genus *Baetiella* but only in 4 out of 21 species, *Baetiella* (*Baetiella*) *bispinosa* (Gose 1980), *B.* (*B.*) *subansiri*  Vasanth, Selvakumar, & Subramanian 2020, *B. macani* (Müller-Liebenau, 1985) and *B.* (*B.*) *bibranchia* Phlai-Ngam, Boonsoong, Gattolliat, & Tungpairojwong 2024. While in 2 other genera, *Branchiobaetis* and *Asiobaetodes*, the presence of accessory gills was considered to be a diagnosis character. The genus *Branchiobaetis* comprises 7 species, and the accessory gills are presented in 2 positions: coxal gill and maxillary gill. In the monotypic genus *Asiobaetodes*, the accessory gills are the coxal gill and labium gill.

Additionally, the presence of the accessory gill on winged stages of Ephemeroptera is extremely rare. It was mentioned only in 4 genera. Three genera, *Murphyella* Lestage, 1930, *Coloburiscoides* Lestage, 1935, and *Coloburiscus* Eaton, 1888 (family Coloburiscidae), occur in the Neotropical, Australian, and New Zealand regions, while 1 genus, *Isonychia* Eaton, 1871 (family Isonychiidae), is found in Europe. Štys and Soldán (1980) also stated that accessory gills of the apomorphic suborder Schistonota (Baetidae, Oligoneuriidae: Chromarcyinae, Origuneuriinae) are not retained by the adults.

Herein, we described a new monotypic genus from Thailand with the data provided for all stages. This is the first genus of Baetidae with accessory gills observed in both larval and winged stages. Molecular data (COI) are also provided for the further studies. Generic position is discussed based on all stages. The comparison with the larval morphology of the presumably related genera is provided. The presence of accessory gills in Baetidae from around the world is also summarized, and discussion on accessory gills on wing stages of the order Ephemeroptera is provided.

## Materials and Methods

Specimens were collected from the Mae Chaem River, Chiang Mai, Thailand between December 2024 and March 2026. Baetid larvae were collected by kicking method in a flowing area with D-frame nets. Mature nymphs were kept in an earthenware pot which had net cover and maintained adequate oxygen until the emergence of subimago. Subimago was kept in net cover through the following day when molting to imago. Other specimens were preserved in 99% ethanol. In addition, some live specimens were sorted, photographed, and video recorded in the field using iPhone15 Pro max (video setting: 4K at 30 fps) (Apple, Inc.). Selected larvae and exuviae from rear adult stages belonging to the new taxon were dissected and mounted on slides in Hoyer’s mounting medium. Two live specimens were dyed with methylene blue for 10 min then moved to fix in glycerol for visualizing the tracheal system (Lorenzen and Gary 1986).

Photograph of characters and habitus were taken using an Olympus EP50 microscope. Photographs of habitus were taken using NIKON SMZ445 and Canon EOS 6D camera and the Visionary Digital Passport imaging system (http://www.duninc.com) and processed with Adobe Photoshop Lightroom (http://www.adobe.com) and Helicon Focus version 5.3 (http://www.heliconsoft.com). For scanning electron microscopy (SEM), specimens were dehydrated with a graded series of ethanol, critical point dried in CO_2_ in Quorum Technologies CPD7501, then coated with gold with Quorum Technologies SC7620, and viewed with a FEI quanta 450 electron microscope (Scientific Equipment Center, Faculty of Science, Kasetsart University, Bangkok, Thailand).

Final plates were generated using Adobe Photoshop 2020. The material examined is housed in the Zoological Museum at the Veterinary Anatomy and Pathology Museum Chiang Mai University (VMCMU), Chiang Mai, Thailand, the Aquatic Insects Collection of the Zoological Museum Kasetsart University (ZMKU), Bangkok, Thailand and Naturéum, Muséum des Sciences Naturelles (MZL), Lausanne, Switzerland.

DNA was extracted using non-destructive methods to allow subsequent morphological analysis (see Vuataz et al. 2011 for details). Total DNA was extracted using a Nucleospin Tissue (NucleoSpin, Macherey-Nagel, Germany) following the manufacturer’s protocol. The COI amplification was performed using LCO1490 and HCO2198 (Folmer et al. 1994). The polymerase chain reaction (PCR) conditions and procedure was set as: 300 s at 94 °C; 30 s at 94 °C, 30 s at 52 °C, 50 s at 72 °C (×40 cycles); 600 s at 72 °C. The PCR products were sent to purified sequenced by U2Bio Co., Ltd (Korea). The GenBank accession numbers are given in Table 1, nomenclature of gene sequences follows Chakrabarty et al. (2013). Nucleotide sequences obtained in this study have been deposited in GenBank. The distribution map was generated with the SimpleMappr software (https://simplemappr.net; Shorthouse 2010).

**Table 1.** Sequenced specimens of *Gattollius* gen. nov. *et* sp. nov.

Species	Locality	Code	GenBank #	GenSeq nomenclature
***Gattollius thailandicus* gen. nov. *et* sp. nov.**	Mae Chaem	GATTO01	PX655555	genseq-2 COI
	Mae Chaem	GATTO02	PX655556	genseq-2 COI

We followed all guidelines of the Animal Ethics Committee of Kasetsart University (approval no. ACKU68-SCI-017) and the Faculty of Veterinary Medicine Chiang Mai University Animal Care and Use Committee (Approval no. FVMCMU-ACUC R11/2569) for collecting the mayfly specimens.

## Results

***Gattollius* gen. nov. Suttinun & Boonsoong, 2026** 

urn:lsid:zoobank.org:act:34D8FC90-EF37-4A19-8E96-43C57914E7B1

**Fig. 1.**
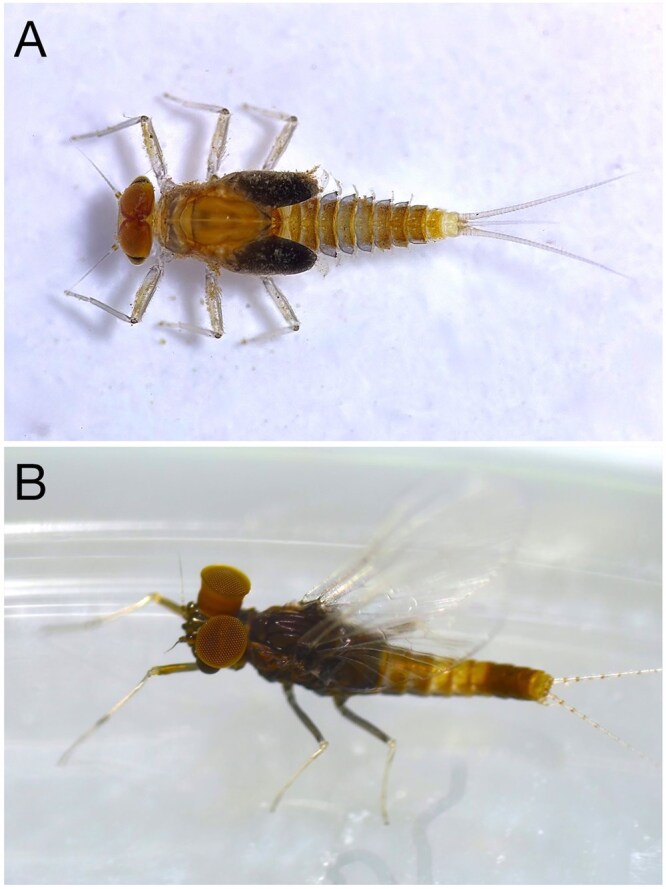
*Gattollius thailandicus* gen. nov. *et* sp. nov., live habitus: A) male larva, B) male imago.

**Fig. 2.**
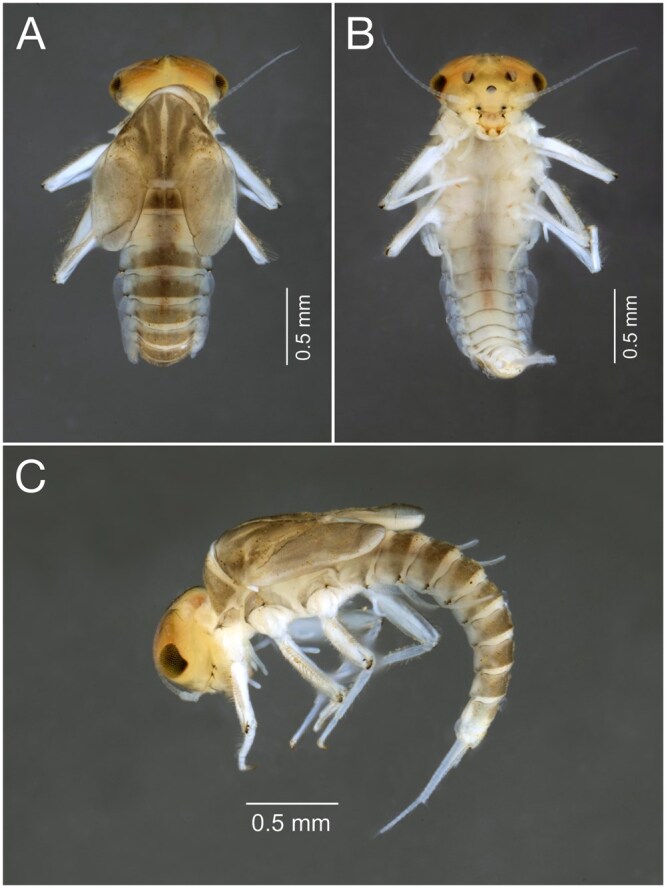
*Gattollius thailandicus* gen. nov. *et* sp. nov., male larva habitus: A) dorsal view, B) ventral view, C) lateral view.

**Fig. 3.**
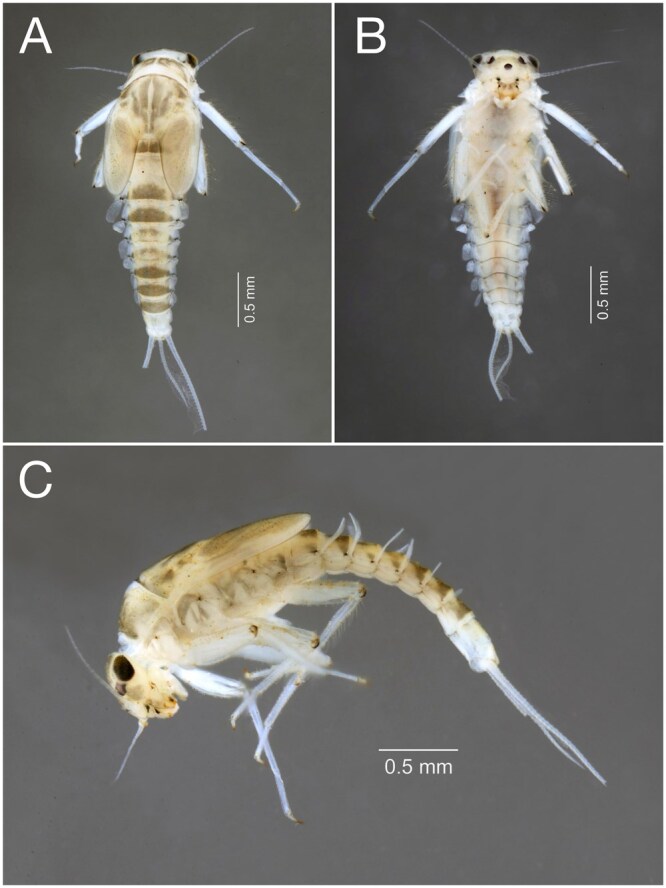
*Gattollius thailandicus* gen. nov. *et* sp. nov., female larva habitus: A) dorsal view, B) ventral view, C) lateral view.

**Type species.** *Gattollius thailandicus* sp. nov. (Figs 1–3, 5–7), by present designation

### Diagnosis

#### Larva

**Fig. 4.**
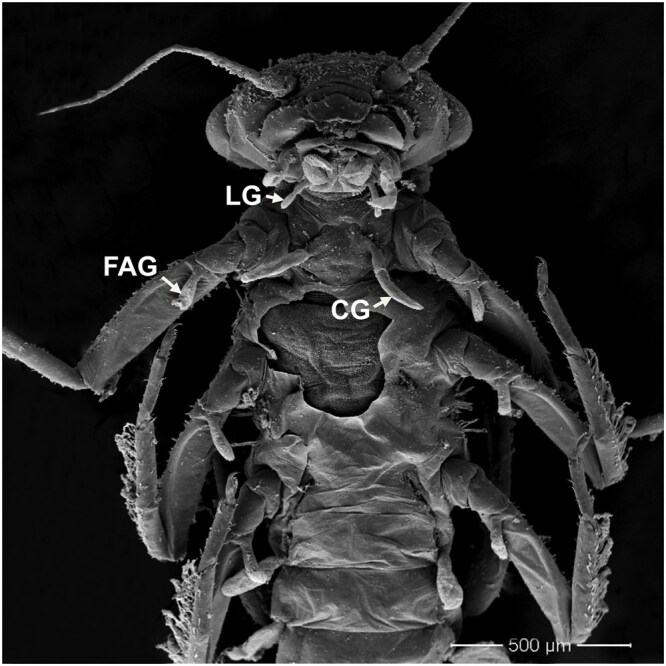
*Gattollius thailandicus* gen. nov. *et* sp. nov., SEM of male larva, ventral view. LG, labium gills; CG, coxal gills; FVG, femoral villopore gills.

(i) Body (Figs 1A, 2, and 3) ventrally flattened. (ii) Accessory gills (Fig. 4) present in 3 positions: at base of mentum, between coxa and prosternum and plate-like gill within femoral groove. (iii) Mandibles (Fig. 8A-C) with incisors largely fused; left mandible with subtriangular process above mola; right mandible with straight prostheca–mola margin. (iv) Hindwing pads absent. (v) Femur (Figs 9 and 10) with distinct rows of multiciliate setae. (vi) Seven pairs of gills (Fig. 11) on segments I to VII; posterior margins of terga with stout triangular spines. (vii) Gonostyli buds (Fig. 12B) *Acentrella-*type. (viii) Median caudal filament (Figs 1A and 3) developed.

#### Imago

**Fig. 5.**
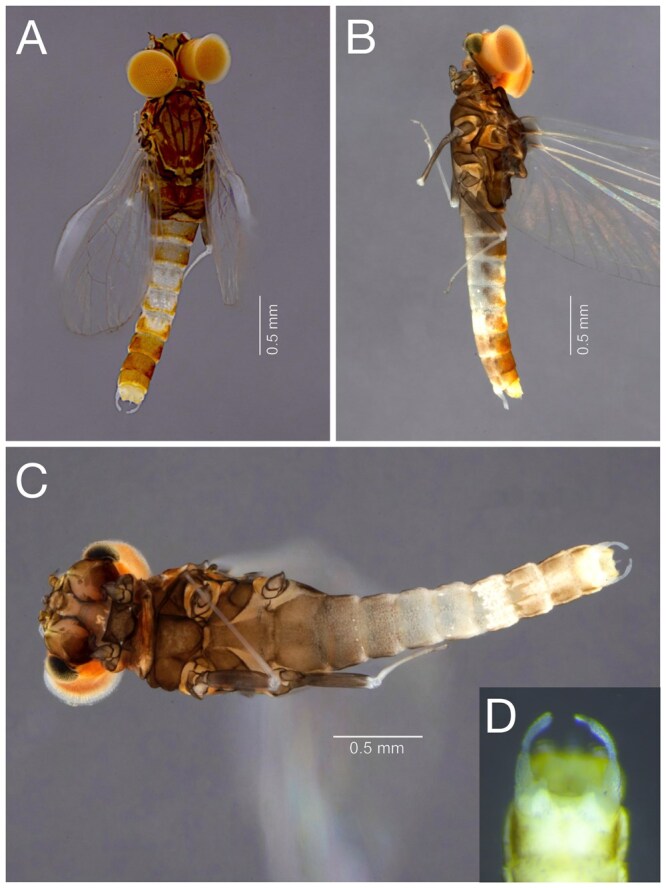
*Gattollius thailandicus* gen. nov. *et* sp. nov., male imago habitus: A) dorsal view, B) lateral view, C) ventral view, D) male forceps ventral view.

**Fig. 6.**
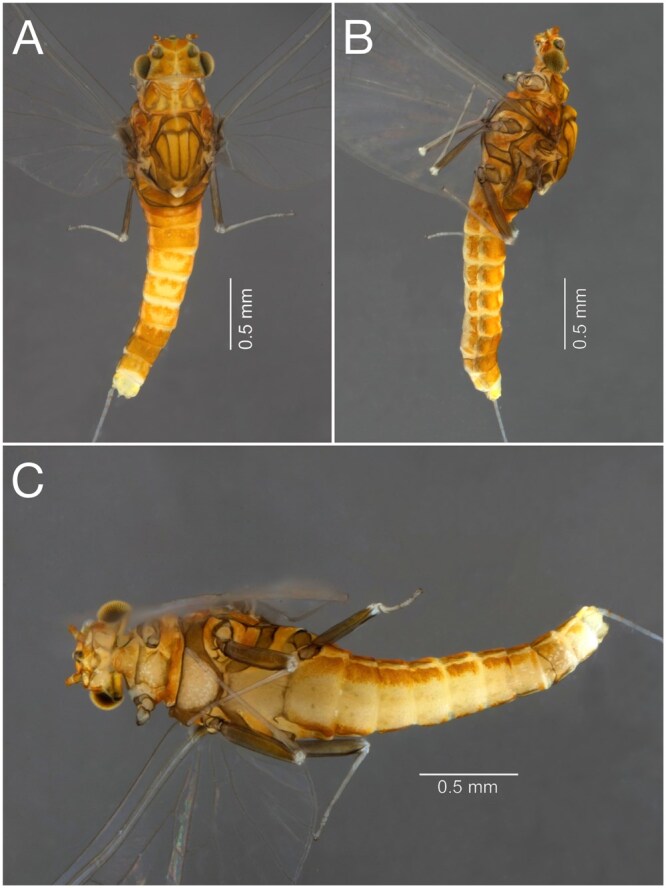
*Gattollius thailandicus* gen. nov. *et* sp. nov., female imago habitus: A) dorsal view, B) lateral view, C) ventral view.

(i) Prosternum (Figs 5, 6, 13A, and 13B) with a tiny remnant of finger-like thoracic gills at the area between coxa and sternal plate. (ii) Mesothorax (Figs 5 and 6) darker, infrascutellum (ISL) with membranous extension. (iii) Anteronotal protuberance (Figs 5 and 6) conic and sharply projected upward. (iv) Marginal intercalary veins (Fig. 12A) paired until MP1. (v) Hindwings (Figs 5 and 6) absent. (vi) Male genitalia 3 segmented, curved, apically round (Fig. 5D).

#### Subimago

**Fig. 7.**
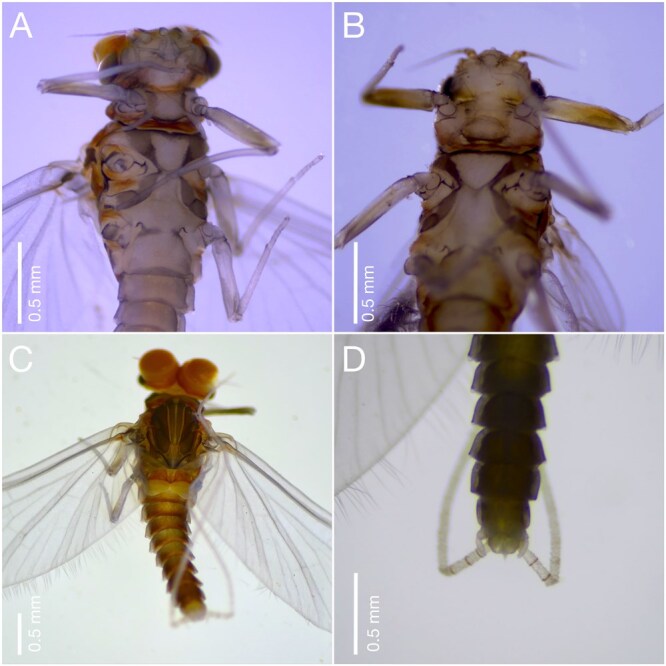
*Gattollius thailandicus* gen. nov. *et* sp. nov., subimago: A) male head and thorax ventral view, B) female head and thorax ventral view, C) male dorsal view, D) male forceps ventral view.

**Fig. 12.**
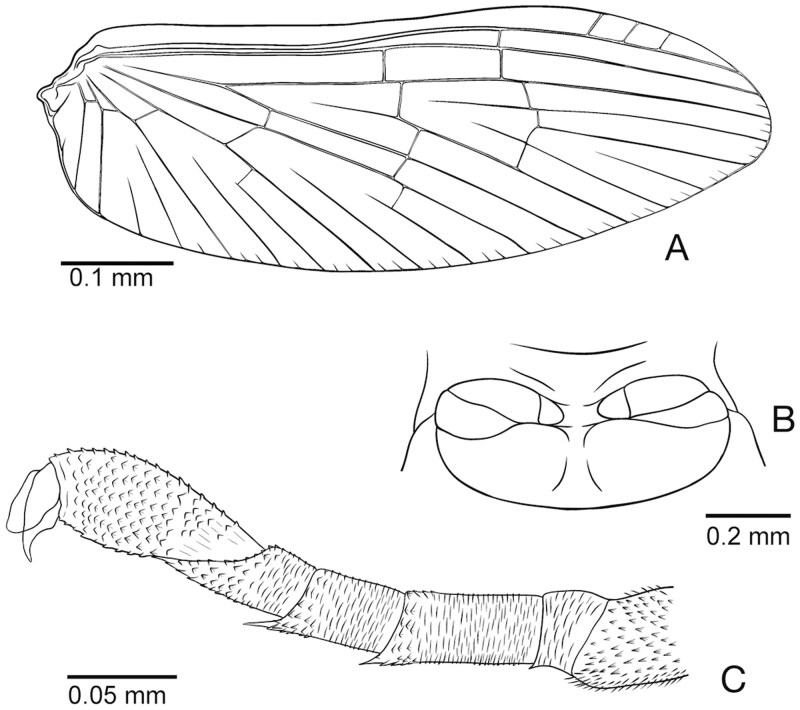
*Gattollius thailandicus* gen. nov. *et* sp. nov.: A) female imago forewing, B) male larva gonostyli bud *Acentrella*-type, C) subimago tarsus of foreleg.

**Fig. 13.**
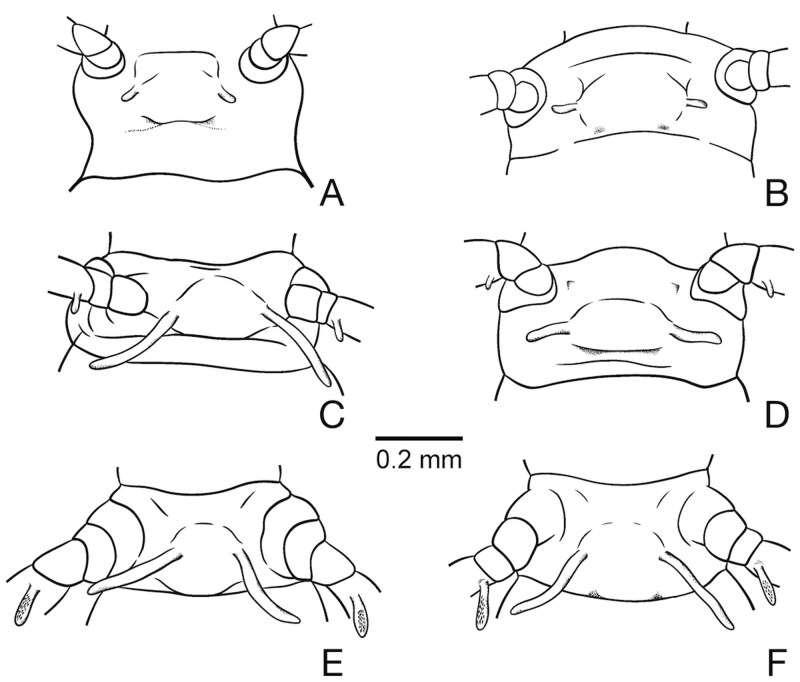
*Gattollius thailandicus* gen. nov. *et* sp. nov.: A) accessory gills of male imago, B) accessory gills of female imago, C) accessory gills of male subimago, D) accessory gills of female subimago, E) accessory gills of male larva, F) accessory gills of female larva.

(i) Prosternum (Fig. 7A, 7B, 13C, and 13D) with a remnant of finger-like thoracic gills at the area between coxa and sternal plate. (ii) Legs (Fig. 7A, 7B, 13C, and 13D) with a remnant of plate-like accessory gills at proximal of femur. (iii) Tarsal (Fig. 12C) segment V covered mainly by pointed microlepides (not blunt). (iv) Hindwings (Fig. 7) absent.

### Description

**Imago** (Figs 5 and 6)

**Head** (Figs 5 and 6). Light brown; antenna with basal segments light brown and flagellum paler; female eyes dark; male eyes orange.

**Thorax** (Figs 5 and 6). Generally light brown to darkened on mesothorax. Prothorax light brown; small remnant of thoracic gill (Fig. 13A and 13B) present between coxa and sternum. Mesothorax darker, with membranous extension of ISL and a distinct anteronotal protuberance. Legs pale, femora darker, femoral accessory gill absent in this stage. Forewing (Fig. 12A) with pale venation and reduced crossveins in stigmatic area; marginal intercalaries paired. Hindwings absent.

**Abdomen** (Figs 5 and 6). Predominantly whitish with orange markings on anterior and lateral terga and sterna. Caudal filaments translucent with darker band on each segment, longer than body.

**Male Genitalia** (Fig. 5D). Basal segment subquadrate; second segment curved and medially constricted; third segment elongate, medially curved, and apically rounded.

**Fig. 14.**
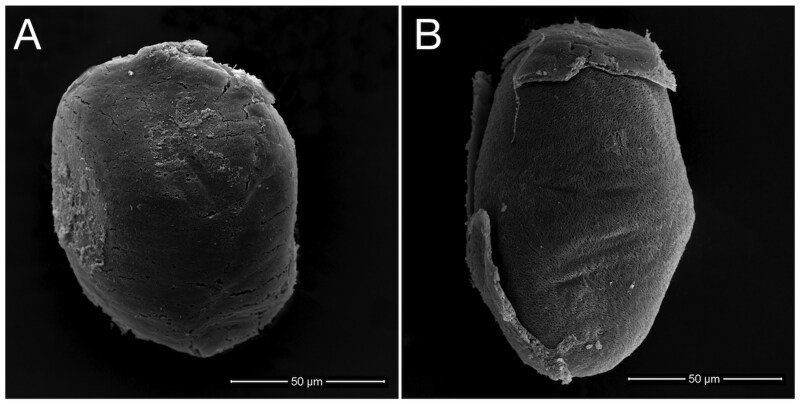
*Gattollius thailandicus* gen. nov. *et* sp. nov., SEMs of egg: A) with chorion membrane, B) without chorion membrane.

**Eggs** (Fig. 14) Irregularly oval; surface of chorion rugose.

**Subimago** (Fig. 7)

**Head** (Fig. 7). Light brown; turbinate portion of compound eyes orange in male; eyes dark in female. Antenna with basal segments light brown and flagellum paler.

**Thorax** (Fig. 7). Generally light brown to darkened on mesothorax; ISL with membranous extension. Prosternum with remnant of thoracic gill between coxa and sternum. Legs pale, with remnant of plate-like femoral accessory gill present (Fig, 13C and 13D); tarsi (Fig. 12C) covered with mainly by blunt microlepides, segment V covered with pointed microlepides. Forewing with pale venation and paired marginal intercalaries; hindwing absent.

**Abdomen** (Fig. 7). Terga predominantly greyish, terminal segment paler; sterna whitish. Caudal filaments pale. Male genitalia with 3-segmented forceps.

**Larva** (Figs 1–3).

**Fig. 15.**
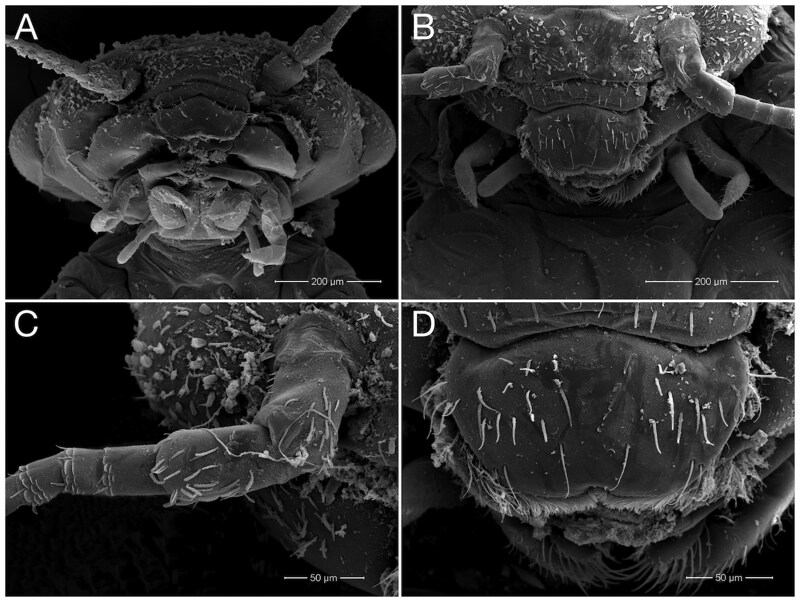
*Gattollius thailandicus* gen. nov. *et* sp. nov., SEMs of head: A) head (frontal view), B) anterior of head (dorsal view), C) antennal scape and pedicel, D) labrum (dorsal view).

**Antenna** (Figs 4 and 15). Multi-segmented; segments with simple setae.

**Labrum** (Fig. 15D). Rectangular, broader than long; anteromedian notch shallow with a small, rounded lobe. Dorsal surface bearing simple setae along lateral and distal margins; ventral surface with feathered setae laterally.

**Fig. 8.**
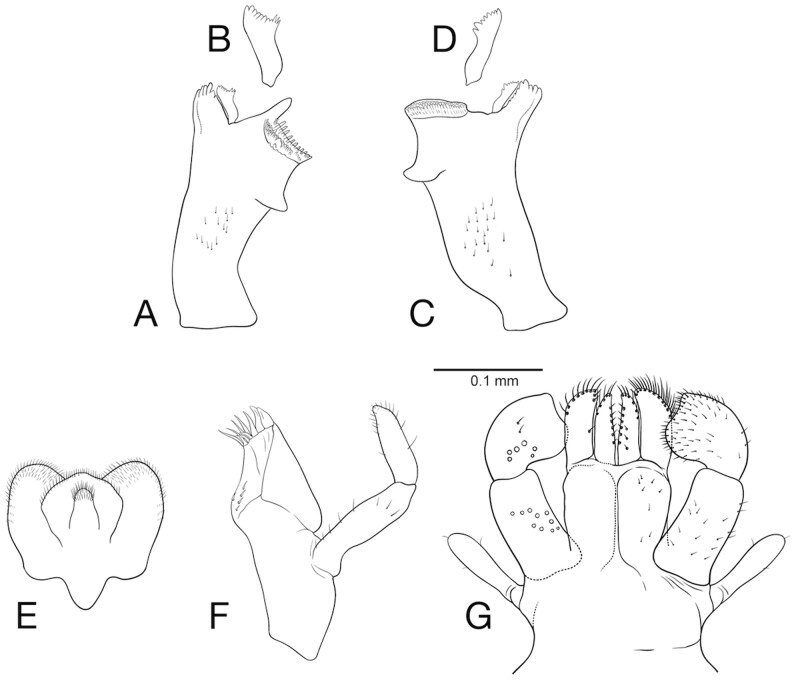
*Gattollius thailandicus* gen. nov. *et* sp. nov., mouthpart morphology: A) left mandible, B) left prostheca, C) right mandible, D) right prostheca, E) hypopharynx, F) maxilla, G) labium.

**Mandibles** (Fig. 8A-D). Incisors largely fused, apically rounded; inner margin denticulate. Prostheca denticulate apically; left mandible with well-developed subtriangular process above mola; right mandible with straight margin between prostheca and mola.

**Hypopharynx** (Fig. 8E). Lingua and superlingua subequal; margins with dense setae.

**Maxilla** (Fig. 8F). Galea-lacinia with denti-setae in dorsal row. Maxillary palp 2-segmented, segment II shorter and apically pointed.

**Labium** (Fig. 8G). Labial palp 3-segmented, segment I longest, segment II and III completely fused, outer margin curved apically straight. Glossae and paraglossae with simple and pectinate setae. Accessory gill oblong, present at base of mentum; tracheation well developed.

**Thorax** (Figs 9, 10, and 16-19). Hindwing pads absent. Femur with distinct rows of specialized setae and bearing a plate-like accessory gill within a femoral groove; thoracic accessory gill present between coxa and prosternum. Tibia and tarsus with rows of simple setae; patella-tibial suture present; tarsus without a subapical seta; tarsal claw denticulate.

**Abdomen** (Figs 11 and 20). Terga with posterior triangular spines. Seven pairs of abdominal gills present, gill I ventrally oriented; tracheation developed. Gonostyli buds (Fig. 12C) *Acentrella*-type. Paraproct (Fig. 21) without posterior projection. Cerci long; median caudal filament shorter than cerci.

**Distribution**. Thailand (Fig. 22).

### Etymology

The name of this genus is dedicated to Dr. Jean-Luc Gattolliat (Naturéum, Muséum des Sciences Naturelles, Lausanne) for his outstanding contributions to the Systematics of the family Baetidae in Southeast Asia and more recently in Thailand. The gender is masculine.

***Gattollius thailandicus* sp. nov. Suttinun & Boonsoong, 2026** 

urn:lsid:zoobank.org:act:53EB6547-CCE3-4A0C-9492-333475A845B0

### Material Examined

**HOLOTYPE:** One larva in ethanol, 1 female imago in ethanol, 1 female sub-imago in ethanol, 1 male sub-imago in ethanol (VMCMU). THAILAND, Chiang Mai province, Mae Cheam district, Mae Chaem River, Mae Chaem weir, 18°29′34.1″N 98°21′50.1″E, 467 m, 15.XII.2024, C. Suttinun leg.; 1 male imago in ethanol, same locality, 31.I.2026, C. Suttinun leg. **PARATYPES:** 2 larvae on slides and 18 larvae in ethanol (VMCMU) same data as holotype; 2 larvae in slide (ZMKU), same data as holotype; 2 larva in ethanol, same data as holotype (ZMKU); 6 larvae in ethanol, 2 female sub-imago (VMCMU) same locality, 26.I.2025, C. Suttinun leg; 2 larvae in ethanol, 1 female sub-imago (MZL) same locality, 26.I.2025, C. Suttinun leg; 6 larvae in ethanol (VMCMU) same locality, 31.I.2026, C. Suttinun leg; 12 larvae in ethanol (VMCMU) same locality, 27.III.2026, C. Suttinun leg.

**Male Imago** (Figs 1B and 5)

**Length.** Body 2.0 mm (*n* = 1).

**Head** (Fig. 5). Hypognathous, light brown, length 0.5× maximum width. Scape, pedicel brown and flagellum whitish. Turbinate eyes orange; inner margins with space between them.

**Thorax** (Fig. 5). Base brown. Prothorax light brown; prosternum (Figs 5 and 13A) with a tiny remnant of finger-like thoracic gills at the area between coxa and sternal plate, length 0.2× length of the larval length. Mesothorax blackish, ISL with membranous extension, anteronotal protuberance conic and sharply projected anterior-dorsal. Legs whitish except femur greyish; remnant of plate-like accessory gills at the proximal of femur absent; Ratios of foreleg femur: tibia: T1+T2:T3:T4:T5 = 1:1.56:0.41:0.24:0.12:0.17. Forewing (Fig. 12A) with whitish veins; stigmatic area with 2 cross veins reaching subcostal vein; marginal intercalary veins paired until MP1. Hindwings absent.

**Abdomen** (Fig. 5). Terga I to III orange; terga IV to VI light orange with orange area on posterolateral margins; terga VII to IX orange; tergum X whitish. Sterna whitish without distinct marking. Caudal filament transparent with orange strip on each segment until mid-length.

**Genitalia** (Fig. 5D). basal segment subquadrate; segment II with overall curved appearance, strongly constricted medially and slightly widen at the end; segment III length twice its wide, curved medially, round apically.

**Female Imago** (Fig. 6)

**Length.** Body 2.3 mm (*n* = 2).

**Head** (Fig. 6) Light brown, length 0.5× maximum width. Scape, pedicel light brown and flagellum whitish. Eyes black.

**Thorax** (Fig. 6) Base light brown. Prothorax light brown; prosternum (Figs 6 and 13B) with a tiny remnant of finger-like thoracic gills at the area between coxa and sternal plate, length 0.2× length of the larval length. Mesothorax blackish, ISL with membranous extension, anteronotal protuberance conic and sharply projected anterior-dorsal. Legs whitish except femur greyish; remnant of plate-like accessory gills at the proximal of femur absent. Forewing (Fig. 12A) with whitish veins; stigmatic area with 3 cross veins reaching subcostal vein; marginal intercalary veins paired until MP1. Hindwings absent.

**Abdomen** (Fig. 6) Whitish, tergum I orange, terga II to VI whitish with a pair of orange maculae laterally, tergum X whitish. Sterna I to IX whitish with a pair of orange maculae laterally, sternum X whitish. Caudal filament transparent, length 1.5× of body length.

**Eggs** (Fig. 14) Irregularly oval and surface of chorion rugose.

**Male subimago** (Fig. 7A, C, and D)

**Length.** Body 3.0 mm (*n* = 1).

**Head** (Fig. 7A and C). Light brown. Turbinate portion of compound eyes orange. Scape, pedicel light brown and flagellum whitish.

**Thorax** (Fig. 7A and C). Base light brown. Prothorax light brown; prosternum with remnant of finger-like thoracic gills at the area between coxa and sternal plate, same length as the larval length. Mesothorax blackish, ISL with membranous extension. Legs whitish with the remnant of plate-like accessory gills (Figs 7A and 13C) at the proximal of femur, length 0.5× the larval length; tarsal (Fig. 12B) segment I to IV covered mainly by blunt microlepides, segment V covered with pointed microlepides. Forewing with whitish veins; stigmatic area with 3 cross veins reaching subcostal vein; marginal intercalary veins paired. Hindwing absent.

**Fig. 23.**
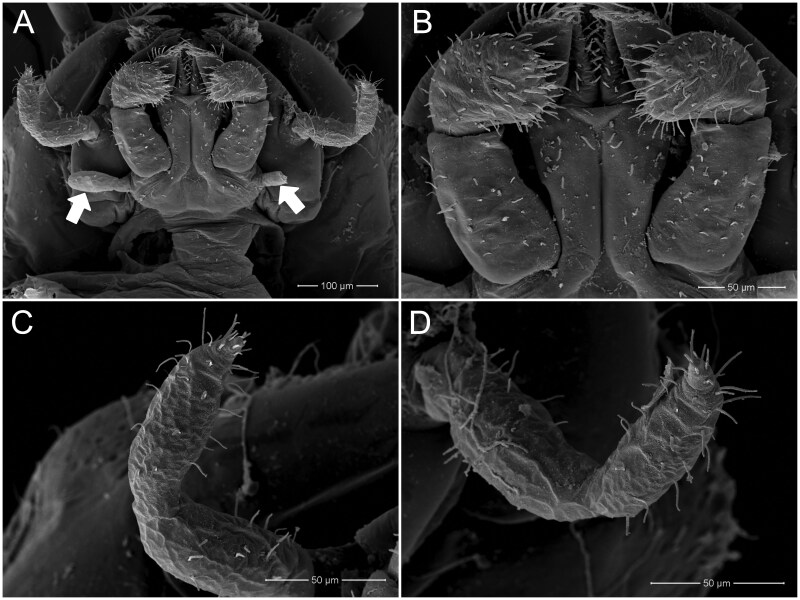
*Gattollius thailandicus* gen. nov. *et* sp. nov., SEMs of mouthpart (ventral view): A) labium and maxilla (ventral view), accessory gills (LG—arrow); B) labium (ventral view); C) right maxillary palp (ventral view); D) left maxillary palp (ventral view).

**Abdomen** (Fig. 7A and C). Terga I to IX uniformly greyish, terga X whitish; sterna uniformly whitish. Caudal filaments whitish. Genitalia with forceps 3-segmented whitish (Fig. 7D).

**Female subimago** (Fig. 7B). Coloration as in male subimago except the remnant of finger-like thoracic gills (Figs 7B and 13D) length 0.7× of the larval length.

**Length.** Body 3.0 mm (*n* = 3).

**Larva** (Figs 1A, 2, and 3). Body length 2.5 to 2.9 mm.

**Coloration** (Figs 1–3). Head dorsally light brown. Thorax dorsally dark brown with a few diffusive light orange maculae on pleura medially and a few diffusive blackish close to median suture. Abdomen terga I to IX light brown, terga X yellow. Head and thorax ventrally yellow, abdomen ventrally yellowish with apical area of each sternum darker. Legs whitish. Caudal filaments yellowish.

### Head

**Antenna** (Figs 4 and 15). Approximately 18 segments; Scape and pedicel (Fig. 11C) (Fig. 15C) with fine, simple setae scattered over surface. Flagellum with blunt scale and fine, simple setae at apical area of each segment.

**Labrum** (Fig. 15B and D). Rectangular, length 0.6× maximum width. Anteromedian notch shallow with a small, rounded lobe at the base. Dorsal surface with long, fine, simple setae erratically scattered and with an arc of 8 stout, simple setae along lateral margin; an arc of 4 to 5 long, fine, simple setae at basal area projection laterally; an arc of 4 to 6 stout, simple setae anteromedially along distal margin. Ventral surface with feathered setae along the anterior margin laterally.

**Left mandible** (Fig. 8A and B). Canine with 4 + 3 denticles apically rounded, largely fused, inner margin of inner incisor with 2 small denticulations. Prostheca apically with small denticles and a comb-shape structure. Subtriangular process long and slender; inner margin straight without teeth, above level of area mola. Mola with well-developed denticulation. Basal surface with long, fine, simple setae scattered.

**Right mandible** (Fig. 8C and D). Canine with 4 + 3 apically rounded denticles, largely fused, inner margin of inner incisor with small denticulation. Prostheca robust, apically with small denticles and comb-shaped structure. Margin between prostheca and mola straight. Mola with developed denticulation; fine setae at apex of mola present. Basal surface with long, fine, simple setae scattered.

**Hypopharynx** (Fig. 8E). Lingua equal to superlingua, longer than broad, club-shaped with point projection covered with long, thin setae. Superlingua distally with a concave margin, with long, fine setae along distal margin; lateral margin rounded with simple setae along lateral margin.

**Maxilla** (Fig. 8F). Galea-lacinia without setae under crown; lateral margin without setae; inner dorsal row of setae with 4 denti-setae; distal denti-seta with 4 tooth-like setae, middle denti-seta with 2 medium, pectinate setae, proximal denti-seta with a row of 4 long, slender, simple setae. Medially with 4 simple setae. Maxillary palp (Fig. 23C and D) 1.9× as long as length of galea-lacinia, 2-segmented; palp segment II 0.7× length of segment I; segment I with a few of fine setae; segment II curved, apically pointed and with a few fine setae.

**Labium** (Figs 8F, 23A, and 23B). Glossae dorsal surface with a row of 3 short, fine setae at basal area; outer margin with 4 medium, simple setae; inner margin with 6 short, simple setae; ventral surface bare; apical with a long, simple seta. Para glossae dorsal surface with a fine, simple seta at inner margin of basal area and a medium, simple seta at inner margin of near apical area; outer margin covered with 2 rows of 8 long, pectinate setae and 6 medium, pectinate setae; apex with a long, pectinate seta; ventrally bare. Labial palp with segment I 1.4× length of segments II and III combined; segment I dorsal surface with chloride cells and fine, simple setae scattered over area; segment II and III outer margin curved apically straight projected closed to glossa with stout, simple setae and fine, simple setae scattered over area. Accessory gill (Figs 1A, 2, 3, 8G, 15A, 15B, and 23A) oblong, finger-like, present at the base of mentum projected laterally each side; accessory gill length 0.8× length of labium palp segments I; surface with a few fine, simple setae scattered; tracheation (Fig. 24B) well developed.

### Thorax

**Hindwing pads.** Absent.

**Foreleg** (Fig. 9A). Ratio of foreleg segments 2.0:2.0:1.0:0.3. *Femur* (Fig. 10) length 3.5× maximum width; outer margin with 2 type of rows, a row of approximately 19 to 20 very long, bipectinate setae and a row of approximately 14 short, spatulate setae; dorsal surface with a row of 6 medium, spatulate setae near outer margin and a few club-shaped setae and thin, simple setae scattered; inner margin with a row of approximately 14 short, spatulate setae; a groove of femoral (Figs 16A and 17A) at the inner margin presented extending from proximal to distal margins containing a plate-like accessory gill (Figs 4, 13E, 13F, 16, 17, 19B, and 19D) at proximal area and numerous chloride cells present on groove surface covering 2/3 of femur length; accessory gill plate-like, outer surface bears femoral patch (Figs 16, 17, 19B, and 19D) (villopore) oriented ventrally, tracheation (Fig. 24A and D) well developed and branched; apex of femur (Fig. 10D) with numerous stout setae. *Tibia* (Figs 9A, 18A, and 18B) outer margin with 2 types of rows, a row of approximately 24 long, bipectinate setae and a row of approximately 12 stout, simple setae ending at distal margin with 2 stout, spatulate setae and a tuft of fine, simple setae; patella-tibial suture present at 1/3 of tibia length; inner margin with a row of approximately 8 stout setae ending with 2 short, simple setae and a row of 4 fine, simple setae; surface with fine, simple setae scattered. *Tarsus* (Fig. 18C and D) outer margin with a row of approximately 3 pointed, simple setae increasing in size present at apical and fine setae scattered along margin; inner margin with a row of 4 stout to short, pointed, simple setae increasing in size; surface with fine, simple setae scattered. *Tarsal claw* (Figs 9D, 18C, and 18D) with a row of 14 denticles increasing in length toward apex.

**Thoracic gill** (Figs 2–4, 13E, 13F, and 19). A finger-like accessory gill present at the area between coxa and prosternum; tracheation (Fig, 24A and C) very well developed with a complex branching system.

**Middle leg and hind leg** (Fig. 9B and C). As foreleg.

### Abdomen

**Terga** (Fig. 11H). Shagreen; few very long thin setae scattered over surface. Posterior margin of segment with stout triangular spines.

**Sterna** (Figs 11I and 21A). Distal margin without spines and structure; surface basal area with a patch of notched scales on each side, laterally with chloride cells.

**Gills** (Fig. 11A–G). Seven pairs of gills present on abdominal tergites I to VII. Gill I (Figs 2, 11A, 20A, and 20B) oblong, oriented vernally; margin almost bear; tracheation extending from main trunk to outer margin and most branches in gill I, transparent in absolute ethanol and Hoyer’s medium, visible in methylene blue. Gill II to VII (Fig. 11B–G) oblong, reduced in size, oriented laterally; margin covered with scattered long, fine hair-like setae; tracheation extending from main trunk to almost outer margin and reduced to poor in gill VII consequently, transparent in absolute ethanol and Hoyer’s medium, visible in methylene blue.

**Gonostyli bud** (Fig. 12B). *Acentrella*-type, 3 segments. Segment III 0.4× length of segment II.

**Paraproct** (Fig. 21B–D). Posterior margin bared without prolongation at posterior margin; surface with a patch of notched scales and fine, simple setae scattered. Cercotractor margin and surface bared.

**Caudal filaments** (Figs 1A and 3). Cerci approximately 0.8× body length; median caudal filament approximately 0.5× cerci length.

### Etymology

From the word “Thailand” referred to highlight the first country of discovery.

### Biological Aspects

The specimens were collected at an altitude 460 m a.s.l in shallow border of sandbar in the middle of a large river (Fig. 25). Larvae were found in sandy and pebble bottom substrates, usually together with larvae of *Acentrella* (*Liebebiella*) *vera* (Müller-Liebenau 1982) (Ephemeroptera: Baetidae).

**DNA barcoding.** 658 bp of COI sequences were obtained from 2 specimens from the type locality (Table 1).

## Discussion

### Taxonomic Position

The genus *Gattollius* gen. nov. clearly belongs to the family Baetidae based on the following larval characters: (i) a Y-shaped epicranial suture extending ventrally to the lateral ocelli, (ii) relatively long antennae originating anterolaterally on the head, (iii) developing turbinate eyes in late-instar male larvae, (iv) a labrum with a median incision, (v) a glossa widened basally, and (vi) the shape of the right and left mandibular prosthecae. Additional imaginal characteristics include (i) turbinate eyes in the male subimago and (ii) forewings with intercalary veins (Wang and McCafferty 1996).

This genus can be assigned to the clade Anteropatellata based on the presence of a patella–tibial suture on the foreleg at the larval stage; to the clade Baetovectata based on the presence of double intercalary veins in the wings, subimaginal gonostyli buds folded beneath the larval cuticle, and the second segment of the gonostylus directed medially; and to the clade Baetungulata based on the following characters: (i) the inner margin of the claw bearing a single row of denticles, (ii) gills lacking rhythmic respiratory movement, (iii) a 2-segmented maxillary palp, and (iv) foreleg tarsal segments 1 to 4 of the male subimago covered mainly with blunt microlepides (Kluge and Novikova 2011, Kluge 2004, 2022b).

Based on the available evidence, *Gattollius* is considered to be positioned within Baetofemorata, as it possesses part of the proposed autapomorphies, including villopores (femoral patches) present on all larval femora. Additional characteristics correspond to those of Baetofemorata of unclear phylogenetic status, including: (i) a subimaginal gonostylus developing beneath the cuticle of last-instar male larvae and of the “*Acentrella*-type”; (ii) second and third segments of the gonostylus directed medially; (iii) larval antennal bases not meeting and the frons between them not forming a keel; (iv) a stick-shaped mandibular prostheca, denticulate apically, with the inner margin between the prostheca and mola lacking setae; and (v) larval abdominal terga lacking wide scales (Kluge and Novikova 2011).

*Gattollius* clearly belongs to the tribe Acentrellini, as it exhibits a set of diagnostic characters for the tribe as defined by Kluge and Novikova (2011). Larval characters include: (i) partial fusion of labial palp segments II and III, with segment II lacking a large inner-apical projection; (ii) morphology adapted to inhabiting stones in fast-flowing water, characterized by a short body with a flattened ventral surface, an enlarged thorax with widely separated leg bases, a reduced abdomen, long cerci with reduced swimming setae, and ineffective dorsoventral swimming movements (Supplementary Video S1); (iii) a row of long setae on the outer dorsal margin of the larval tibia; (iv) absence of wide scales on the larval abdominal terga; and (v) a less reduced paracercus, approximately half the length of the cerci. Adult characters include: (vi) a conical anteronotal protuberance in both imago and subimago, sharply projected upward; (vii) complete loss of the hindwing; and (viii) an inner-apical convexity on segment II of the gonostylus.

### Comparative Morphology and Related Genera

Selected larval characteristics in comparison with closely related genera are presented in Table 2. *Gattollius* has a ventrally flattened body similar to *Asiobaetodes*  Gattolliat, 2012; and *Acentrella* Bengtsson, 1912; The maxillary palps are 2-segmented with an apical tip, as observed in some species of *Acentrella*. The labial palp is unique, with segments II and III completely fused, lacking an inner lobe, with a straight inner margin and a pointed apex. Accessory gills associated with the mouthparts in shape and position are as observed in *Asiobaetodes*. The hindwing pad is absent, as in some species of *Acentrella* (Waltz and McCafferty 1987, Kluge and Novikova 2011, Phlai-Ngam et al. 2022a, Tungpairojwong et al. 2022).

**Table 2.** Larval character comparisons of *Gattollius* gen. nov. and related genera from Oriental region

Characters	*Asiobaetodes*	*Baetiella*	*Acentrella*	*Gattollius* gen. nov.
**Body**	Ventrally flattened	Ventrally somewhat flattened	Ventrally flattened	Ventrally flattened
**Labium palp**	The second segment with or without an inner apical lobe, the third segment conical with a tiny apical tip	The second segment with or without an inner apical lobe, the third segment relatively symmetrical or slightly asymmetrical, conical, usually with a small tip at apex	The second segment with weakly developed inner lope, the third segment rounded and slightly truncate	Second and third segments completely fused and apical pointed
**Maxillary palp**	Two-segmented, terminal segment without apical tip	Two-segmented, terminal segment with small tip at apex	Two-segmented, terminal segment with or without apical tip	Two-segmented, terminal segment with apical tip
**Accessory gill associated with mouthparts**	Finger-like single gills at the base of mentum	Absent	Absent	Finger-like single gills at the base of mentum
**Hindwing pad**	Highly reduced	Reduced	Vary	Absent
**Accessory gill associated with thoracic sterna**	Finger-like single gills at membrane between coxae and sterna only on foreleg	Absent or finger-like single gills at membrane between coxae and sterna only on foreleg and midleg or all legs	Absent	Finger-like single gills at membrane between coxae and sterna only on foreleg
**Dorsal margin of femur**	With very long and thin setae	With regular or irregular rows of long, simple or feathered setae	With dense multiciliate setae	With 2 regular rows of multiciliate setae
**Accessory gill associated with leg**	Absent	Absent	Absent	Plate-like single gills at the proximal area of femur of all leg
				
**Ventral femoral patch**	Present	Present	Present	Present on femur accessory gills
**Dorsal margin of tibia**	With a row of long, thin setae	With rows of dense, fine, simple setae	Without or with rows of dense, long, multiciliate setae	With 2 rows of long, bipectinate setae and stout, simple setae
**Preapical seta at tarsus**	Present	Absent	Present	Absent
**Subapical setae at tarsal claw**	Absent	A pair is present	Absent	Absent
**Abdominal gills**	Five pairs ventrally oriented; gill margin smooth without serration and setae	Seven pairs, dorsally oriented; gill margin smooth with a few scattered fine hair-like setae	Seven pairs, dorsally oriented	Seven pairs; gill I ventrally oriented, gill margin smooth; gill II to VII dorsally oriented, gill margin with dense long fine hair-like setae
**Posterior marginal spines**	Smooth with independent triangular denticles	Vary; developed with blunt denticle to absent	Well developed	Well developed; Triangular shaped
**Paracercus**	Reduced to a few segments	Reduced; shorter than half of the cerci, multi-segmented or reduced to one segment	Reduced to a few segments	Long, approximately 0.3× to 0.6× of cerci length
**Distribution**	Borneo	Oriental and East Palaearctic region	Arctogea	Northern Thailand (Chiang Mai)
**References**	Gattolliat 2012	Waltz and McCafferty 1987, Vasanth et al. 2020, Phlai-Ngam et al. 2024	Waltz and McCafferty 1987, Phlai-Ngam et al. 2022a	In this study

Finger-like accessory gills associated with the thoracic sterna are observed exclusively on the forelegs in the new genus and *Asiobaetodes*, whereas in the 4 species of *Baetiella* Uéno, 1931, they are present on the thoracic sterna of all legs or restricted to 2 legs. The dorsal margin of the femur bears 2 regular rows of multiciliate setae, as observed in *Acentrella*. The most distinctive characteristic of *Gattollius* is the presence of an accessory gill associated with the leg. These gills are located in the proximal region of the femur of all legs and feature a femoral patch. A detailed discussion of the accessory gills is provided separately. The dorsal margin of the tibia differs from that of related genera in bearing 2 regular rows of long, stout setae.

The preapical tarsal seta is absent in *Gattollius* and *Baetiella* (Phlai-Ngam et al. 2022a). Subapical setae of the tarsal claw are absent in all genera except *Baetiella* (Waltz and McCafferty 1987, Vasanth et al. 2020, Phlai-Ngam et al. 2022a, 2024). *Gattollius* bears 7 pairs of gills, as in other genera, except *Asiobaetodes*, which has 5 pairs oriented ventrally (Gattolliat 2012). Gill I originates on the sternum and is oriented ventrally. Gill I is oblong, with a smooth margin, and arranged parallel to abdominal sternum II. Gills II to VII are oblong and oriented laterally.

The posterior abdominal margin is well developed, bearing a row of pointed, triangular denticles, similar to those observed in *Acentrella*. In contrast, the condition varies in *Baetiella*, where the denticles may be blunt or absent, and in *Asiobaetodes*, where the margin is smooth with independent triangular ­denticles (Waltz and McCafferty 1987, Vasanth et al. 2020, Phlai-Ngam et al. 2024). The paracercus in other genera is reduced, whereas in *Gattollius* it is less reduced and appears 3-tailed. Considering the distributional ranges of each genus, *Asiobaetis* is currently known only from Borneo, whereas *Baetiella* and *Acentrella* have been recorded from Thailand.

Based on the foregoing discussion, *Gattollius* is most closely related to *Acentrella* among genera occurring in Thailand. Diagnostic characters from larval and adult stages, compared with 2 related species are summarized in Table 3. The maxillary palp is generally similar among the 3 species; however, segment proportions differ. In *Gattollius thailandicus* gen. nov. *et* sp. nov., the first segment is approximately 1.5× the length of the second, whereas in the other 2 species it is about 2× as long. The femoral margin bears dense setae in both *G. thailandicus* and *Acentrella fimbriata* Tungpairojwong, Phlai-Ngam, & Jacobus, 2022, but these are arranged in a regular row in the new species and irregularly in *A. fimbriata*. Preapical setae on the tarsus are absent in both species. The posterior margins of the abdominal terga clearly differentiate the 3 taxa. In *Acentrella* (*Liebebiella*) *vera* (Müller-Liebenau 1982), the margin is smooth with blunt to elongate pointed denticles; in *A. fimbriata*, it is smooth with sparse, minute blunt denticles, whereas in *G. thailandicus*, it bears stout, triangular denticles. Gills II to VII possess dense, long, hair-like marginal setae in both *A. fimbriata* and *G. thailandicus*. The male genitalia are similar, with colorless, sharply angulate gonovectes. The most distinctive character of *G. thailandicus* is the well-developed larval median filament, approximately half the length of the cerci, whereas it is reduced in other Thai representatives of the *Acentrella*. Although the new species was consistently collected sympatrically with *A*. (*L*.) *vera* and is superficially similar, it can be readily distinguished by the presence of 3 tails (Tungpairojwong et al. 2022).

**Table 3.** Selected character comparisons of *Gattollius thailandicus* gen. nov. *et* sp. nov. and related species from Thailand (modified from Table 2 of Tungpairojwong et al. 2022)

Characters	*Acentrella (Liebebiella) vera*	*Acentrella fimbriata*	*Gattollius thailandicus* gen. nov. *et* sp. nov.
**Maxillary palp**	First segment length 2 times second segment length, second segment with apical tip	First segment length 2 times second segment length, second segment with apical tip	First segment length 1.5 times second segment length, second segment with apical tip
**Row of setae on margin of femur**	Without irregular row of long setae	Dense, irregular row of long setae present	Dense, 2 regular rows of very long, bipectinate setae and long, spatulated setae
**Preapical setae on tarsal**	One greatly elongated seta present	Absent	Absent
**Metatibial bristles**	Two rows, multilaterally ciliated	Two rows, multilaterally ciliated	Two rows, multilaterally ciliated
**Supapical setae on tarsal claw**	Very small	Absent	Absent
**Posterior margins of terga**	Smooth with blunt to long pointed denticles	Smooth with sparse tiny blunt denticles	Smooth with stout triangular denticles
**Gill margin**	Smooth with scattered short, fine setae	Gills II to VII smooth with dense long, hairlike setae	Gills II to VII with dense long, hairlike setae
**Median filament**	Reduced (approximately 10 to 14 segments)	Reduced (approximately 5 to 7 segments)	Long, approximately 0.3× to 0.6× of cerci length
**Gonoveces of male genitalia**	Colorless, very thin, long, and sharply angulate, with characteristic undulation	Colorless, sharply angulate	Colorless, sharply angulate
**Distribution in Thailand**	Northern and Southern Thailand	Northern Thailand	Northern Thailand (Chiang Mai)
**Biological aspect**	Fast flowing water in varies habitat type range from headwater stream to river	Slow flowing sreams, under the cobble and covered by much sediment	Rim of flowing water in a large river
**References**	Tungpairojwong et al. 2022	Tungpairojwong et al. 2022	This publication

### Accessory Gills in Baetidae and Their Expression in *Gattollius*

The most interesting characteristic of *Gattollius* gen. nov. is the presence of accessory gills in all stages. Accessory gills are rare in Ephemeroptera; an overview and phylogenetic discussion were provided by Štys and Soldán (1980), Staniczek (2010), Zhou (2010), and Kaltenbach et al. (2022b). Here, we provide a comprehensive overview of Baetidae species with accessory gills based on the original descriptions of each species (Table 4). Accessory gills have been reported in 59 species of Baetidae belonging to 10 genera and only in the larval stage. Three types of accessory gills can be distinguished according to their location.

**Table 4.** Position of accessory gills in larva of family Baetidae species (modified from Zhou 2010)

Species	Accessory gills associated with				Distribution	Lineage-based nomenclature	References
	Coxae or thoracic sterna	Maxillary	Labium	Femur			
***Afrobaetodes berneri* Demoulin, 1970**	Single gill located in the membrane between forecoxae and sterna at the base of the forelegs	Single gill at apex of basal segment of maxillary palp	–	–	Afrotropical Region	Anteropatellata = Baetis/fg5 (Kluge and Novikova 2011)	Gattolliat and Sartori 1999 Zhou 2010
***A. lennae* Gattolliat & Sartori, ** 1999							
***Asiobaetis eloi*** Gattolliat, 2012	Single gill located in the membrane between forecoxae and sterna at the base of the forelegs	–	Single gill at base of mentum of labium	–	Oriental Region	Acentrellini (Gattolliat 2012) Baetofemorata	Gattolliat 2012
***Baetiella (Baetiella) bispinosa* (Gose, 1980)**	Single gill located in the membrane between forecoxae and sterna at the base of all legs	–	–	–	Oriental Region	Baetosternata (Kluge and Novikova 2011)	Zhou 2010, Shi and Tong 2015, Vasanth et al. 2020, Phlai-Ngam et al. 2024
***B. (B.) subansiri* Vasanth, Selvakumar & Subramanian, ** 2020							
***B. macani* (Müller-Liebenau, 1985)**							
***B. (B.) bibranchia* Phlai-Ngam, Boonsoong, Gattolliat & Tungpairojwong, 2024**	Single gill located in the membrane between forecoxae and sterna at the base of the forelegs and midlegs	–	–	–			

***Baetodes huaico*** Nieto, 2003	Double gill at apex of coxae in membrane between coxae and trochanters all legs	–	–	–	Neotropical Region	Baetungulata = Baetis/fg7 (Kluge and Novikova 2011)	Mayo 1968, 1972, 1973, Nieto 2004, Zhou 2010
***Ba. chilloni*** Mayo, 1972							
***Ba. gibbus*** Nieto, 2003							
***Ba. pehuenche*** Nieto, 2003							
***Ba. pseudogibbus*** Nieto, 2003							
***Ba. yuracare*** Nieto, 2003							
***Ba. spinae*** Mayo, 1968							
***Ba. proiectus*** Mayo, 1973							
***Ba. levis*** Mayo, 1968							
***Ba. andamagensis*** Mayo, 1972							
***Ba. uruguai*** Nieto, 2003	Single gill at apex of coxae in membrane between coxae and trochanters on all legs	–	–	–			
***Ba. sancticatarinae*** Mayo, 1972							
***Ba. peniculus*** Mayo, 1973							
***Branchiobaetis aduncus* Kaltenbach, Kluge & Gattolliat, 2022**	Single gill located in the membrane between forecoxae and sterna at the base of the forelegs	Single gill at outside of stipes and cardo of maxillary	–	–	Oriental Region	Baetofemorata (Kaltenbach et al. 2022b)	Kaltenbach et al. 2022b
***Br. hamatus* Kaltenbach, Kluge & Gattolliat, 2022**							
***Br. javanicus* (Ulmer, 1913)**							
***Br. jhoanae* Kaltenbach, Kluge & Gattolliat, 2022**							
***Br. joachimi* Kaltenbach, Kluge & Gattolliat, 2022**							
***Br. minangkabau* Kaltenbach, Kluge & Gattolliat, 2022**							
***Br. sabahensis* (Müller-Liebenau, 1984)**							

Species	Accessory gills associated with				Distribution	Lineage-based nomenclature	References
	Coxae or thoracic sterna	Maxillary	Labium	Femur			
***Corinnella thomasi* Boldrini & Salles, 2013**	–	Single gill at the base of the second segment of the maxillary palp	–	–	Neotropical Region	–	Dominique et al. 2002, Boldrini et al. 2012a, Salles et al. 2017, Nieto et al. 2020
***Co. lourii* Salles, Victoriano, Boldrini & Cabette, 2017**							
***Camelobaetidius janae* Thomas & Dominique, 2000**						Baetungulata (Kluge 2022b)	
***Camelobaetidius anubis* (** Traver & Edmunds, 1968 **)**	Single gill at apex of coxae in membrane between coxae and sterna on forelegs	–	–	–	Nearctic and Neotropical Regions	Baetungulata (Kluge 2022b)	Traver and Edmunds 1968, Mayo 1973, Lugo-Ortiz and McCafferty 1995, Nieto 2003, Jacobus and McCafferty 2005, Salles and Serrão 2005, Salles et al. 2005a 2005b, Thomas and Dominique 2006, Boldrini and Salles 2009, Falcão et al. 2011, Sibaja-Araya and Esquivel 2018
***C. apis*** Nieto, 2003							
***C. billi* Thomas & Dominique, 2000**							
***C. francischettii* Salles, Andrade & Da-Silva, 2005**							
***C. guaycara*** Sibaja-Araya & Esquivel, 2018							
***C. leentvaari* Demoulin , 1966**							
***C. maidu* Jacobus & McCafferty, 2005**							
***C. maranhensis* Salles & Serrao, 2005**							
***C. mathuriae* Dominique & Thomas, 2001**							
***C. patricki* Dominique & Thomas, 2001**							

***C. phaedrus* (** Traver & Edmunds, 1968 **)**							
***C. rufiventris* Boldrini & Salles, ** 2009							
***C. serapis* (** Traver & Edmunds, 1968 **)**							
***C. similis* Lugo-Ortiz & McCafferty, ** 1995							
***C. tuberosus* Lugo-Ortiz & McCafferty, 1999**							
***C. warreni* (** Traver & Edmunds, 1968 **)**							
***C. yacutinga* Nieto, ** 2003							
***C. variabilis*** Wiersema, 1998	Single gill located in the membrane between forecoxae and sterna at the base of the forelegs and midlegs	–	–	–			Wiersema 1998
***C. hamadae*** Salles & Serrão, 2005	Double gill at the base of coxae on foreleg	–	–	–			Salles and Serrão 2005, Boldrini et al. 2012a
***C. juparana* Boldrini & Salles, 2012**							
***Dicentroptilum clandestinum*** Lugo-Ortiz & McCafferty, 2001	Double gill at base of coxae in membrane between coxae and sterna on forelegs	–	–	–	Afrotropical Region	Protoptellata (Kluge 2022b)	Wuillot and Gillies 1994, Lugo-Ortiz and McCafferty 2001
***D. papillosum* Wuillot, 1994**							
***D. spinulosa* (Demoulin, 1970)**							

Species	Accessory gills associated with				Distribution	Lineage-based nomenclature	References
	Coxae or thoracic sterna	Maxillary	Labium	Femur			
***Heterocloeon berneri* (** Müller-Liebenau, 1974 **)**	Single gill located in the membrane between forecoxae and sterna at the base of the forelegs	–	–	–	Nearctic Region	Baetungulata (Kluge and Novikova 2011)	Müller-Liebenau 1974, Morihara and McCafferty 1979, Zhou 2010
***H. frivolus* (McDunnough, 1925)**							
***H. petersi* (Müller-Liebenau, 1974)**							
***H. traverae* (** Müller-Liebenau, 1974 **)**							
***Moribaetis maculipennis* (Flowers, 1979)**	Single gill located in the membrane between forecoxae and sterna at the base of the forelegs	–	–	–	Nearctic Region	Baetungulata (Kluge 2022b)	Waltz and McCafferty 1985, Kluge and Vega 2018
***M. salvini* (Eaton, 1885)**							
***Gattollius thailandicus* gen. nov. *et* sp. nov.**	Single gill located in the membrane between forecoxae and sterna at the base of the forelegs	–	Single gill at base of mentum of labium project laterally	Single gill at apical area of inner margin of femur on each legs	Oriental Region	Acentrellini	In this article

- Coxal gills are observed in 9 of the 10 genera: *Afrobaetodes* Demoulin, 1970; *Asiobaetodes*  Gattolliat, 2012; *Baetiella* Uéno, 1931; *Baetodes* Needham & Murphy, 1924; *Branchiobaetis* Kaltenbach, Kluge, & Gattolliat, 2022; *Camelobaetidius* Demoulin, 1966; *Dicentroptilum*  Wuillot & Gillies, 1994; *Heterocloeon* McDunnough, 1925; and *Moribaetis*  Waltz & McCafferty, 1985 (Traver and Edmunds 1968, Mayo 1968, 1972, Müller-Liebenau 1974, Morihara and McCafferty 1979, Waltz and McCafferty 1985, 1987, Wuillot and Gillies 1994, Lugo-Ortiz and McCafferty 1995, 2001, Wiersema 1998, Gattolliat and Sartori 1999, Nieto 2003, 2004, Jacobus and McCafferty 2005, Salles and Serrão 2005, Salles et al. 2005a, 2005b, Thomas and Dominique 2006, Boldrini and Salles 2009, Zhou 2010, Falcão et al. 2011, Gattolliat 2012, Shi and Tong 2015, Kluge and Vega 2018, Sibaja-Araya and Esquivel 2018, Nieto et al. 2020, Vasanth et al. 2020, Kaltenbach et al. 2022b, Phlai-Ngam et al. 2024). This type of accessory gill is the most common within Baetidae and typically occurs in the region between the coxae and the sterna of the forelegs. In 3 species of *Baetiella*, however, these gills are present on all legs (Gattolliat and Sartori 1999, Zhou 2010, Vasanth et al. 2020, Phlai-Ngam et al. 2024). In 2 species of *Baetiella* and *Camelobaetidius*, accessory gills occur on both the fore- and middle legs (Lugo-Ortiz and McCafferty 1995, Phlai-Ngam et al. 2024). These gills are usually single, except in 2 species of *Camelobaetidius* and 3 species of *Dicentroptilum*, in which they are double (Wuillot and Gillies 1994, Wiersema 1998, Dominique et al. 2002, Zhou 2010). In *Baetodes*, accessory gills are located between the coxae and trochanters on all legs and may be either single or double (Mayo 1968, 1972, Nieto 2004, Zhou 2010, Shi and Tong 2015).
- Maxillary gills can be divided into 2 forms: the maxillary gill and the maxillary palp gill. The maxillary gill is single and located between the stipes and cardo and has been observed exclusively in all species of *Branchiobaetis* (Kaltenbach et al. 2022b). The maxillary palp gill may occur as a single gill at the base of the maxillary palp, as in *Afrobaetodes*, or as a single gill at the base of the second segment of the maxillary palp, as reported in 3 species of *Corinnella* and *Camelobaetidius* (Mayo 1973, Waltz and McCafferty 1987, Dominique et al. 2002, Zhou 2010, Boldrini et al. 2012a, Salles et al. 2017). In the original descriptions of these 3 species, this structure was described as a bifid maxillary palp; however, it was later recognized as a maxillary palp gill and subsequently referenced as such by Nieto et al. (2020).
- A labial gill in the form of a single gill at the base of the mentum has been observed only in the monotypic genus *Asiobaetodes* (Gattolliat 2012).

Across major biogeographic regions, accessory gills have been documented exclusively in the larval stage and in genera placed within distinct higher-level lineages in recent classifications. In the Afrotropical region, accessory gills have been reported in 2 genera. *Afrobaetodes* was assigned to the lineage Anteropatellata (=Baetis/fg5) by Kluge and Novikova (2011), whereas *Dicentroptilum* was placed within Protoptellata in the classification of Kluge (2022b). The Oriental genera *Asiobaetodes*, *Baetiella*, and *Branchiobaetis* were placed within differentiated lineages. In its original description, *Asiobaetodes* was assigned to the tribe Acentrellini, which had earlier been considered part of Baetofemorata (Gattolliat 2012, Kluge and Novikova 2011). *Baetiella* was placed within Baetosternata by Kluge and Novikova (2011). *Branchiobaetis* was positioned within Baetofemorata (Kaltenbach et al. 2022b) and the novel genus, *Gattollius* gen. nov. is assigned to Acentrellini tribe in this article. The Neotropical genus *Baetodes* was placed within Baetungulata (=Baetis/fg7) (Kluge and Novikova 2011) and represents the most species-rich lineage in which accessory gills have been documented, occurring in 13 species, whereas the position *Corinnella* remains unclear. In the Nearctic region, *Moribaetis* was placed within Baetungulata Kluge (*2022b)*, whereas the phylogenetic position of *Heterocloeon* remains unresolved; it is currently treated as part of Baetungulata, although Kluge and Novikova (2011) indicated that its relationships require further consideration because it shares a synapomorphy with Acentrella/fg1. *Camelobaetidius*, reported from both the Nearctic and Neotropical regions, was placed within Baetungulata–non-Baetofemorata by Kluge and Novikova (2011) and Kluge (2022b). The scattered occurrence of accessory gills across unrelated lineages indicates that this character is neither stable nor lineage-defining within Baetidae and suggests multiple independent origins.

In the larva of *Gattollius* gen. nov., accessory gills occur in 3 locations: (i) a single gill at the base of the mentum of the labium; (ii) a single finger-like gill on each side of the prosternum, located in the membranous area between the coxae and the sternum; and (iii) a single gill on the proximal region of each femur. The first 2 types represent expansions of the soft membrane between cuticular surfaces. In contrast, the femoral gill differs structurally from the others. These gills are connected to the tracheal system and contain internal tracheae; therefore, a respiratory function is most probable. Although small relative to the gill, the accessory gills exhibit developed tracheation with several internal branches. In the prosternum, the tracheation is complex and connected to the main thoracic tracheal trunk (Fig. 24). *Gattollius* shows similar accessory gills in shape and position on the labium and coxae to those of *Asiobaetodes* from Borneo; however, femoral gills have not been observed in that genus. The femoral gill is located on the inner proximal region of each femur. Its tracheation is well developed, with branches connected to a tracheal duct of each femur, and its outer surface bears an area resembling a femoral patch (Figs 16 and 17). We propose the term femoral villopore gills (FVG) for these specialized accessory gills. The presence of FVG suggests that they may represent secondary structures complementing the abdominal gills, which are immobile and less branched. Their position further supports this interpretation, as they are situated in the proximal region of the femur and project into the water-flowing space between the body and the substrate. However, the presence of a femoral patch on this accessory gill remains unclear, and its functional significance has yet to be determined. Among previously reported taxa, the apophyses on the forefemur of *Camelobaetidius ipaye*  Nieto, 2003 show positional similarity but differ markedly in both shape and point of origin. In *C. ipaye*, the protuberance arises from the anteriormost apical region of the femur, whereas the leaf-like FVG originate from the apicomedial region. Furthermore, these apophyses are present only on the foreleg, whereas FVG occur on all legs (Boldrini et al. 2012b).

Accessory gills in the adult stage are extremely rare in mayflies. They have been reported in 3 genera from the Neotropical, Australian, and New Zealand regions in the family Coloburiscidae—*Murphyella* Lestage, 1930; *Coloburiscoides* Lestage, 1935; and *Coloburiscus* Eaton, 1888—and in one European genus, *Isonychia* Eaton, 1871 (Isonychiidae) (Štys and Soldán 1980). In the mouthparts, remnants of accessory gills in the adult stage have been observed in these 4 genera; however, in *Gattollius*, labial gills are absent in the adult stage. Thoracic accessory gills in adults have been reported only in *Murphyella needhami* Lestage, 1931. In that species, the subimaginal finger-like gills are approximately one-half to one-third the length of the larval gills. In the imago, these gills are reduced by approximately half (Štys and Soldán 1980). In contrast to our observations in *Gattollius*, a finger-like coxal gill in the subimago is as long as the larval gill in males and approximately two-thirds the length of the larval gill in females. In the imago, the coxal gill is reduced to an oval structure approximately one-sixth the length of the larval gill. In *Isonychia*, accessory gills have been reported at the base of the foreleg, showing a similar reduction from larva to subimago and absence in the imago. As observed for FVG in *Gattollius*, remnants are present on all legs in the subimago but absent in the imago. The presence of accessory gills in the adult stage should be considered nonfunctional. Similarly, the persistence of mouthparts in *Siphlonurus dongxi* Li & Tong, 2023 during the adult stage represents a comparable condition, as mayflies are nonfeeding during the aerial phase (Yang et al. 2023). Such remnant structures can therefore be interpreted as vestigial remains of larval features.

In conclusion, *Gattollius* served as the first genus belonging to family Baetidae where accessory gills in adults were observed. The presence of both larval and adult accessory gills in this novel taxon seems to support that their presence is a convergency characteristic among mayfly taxa. Previous studies of the family Baetidae in Thailand have mainly focused on headwater streams, whereas this study in a large river system resulted in the discovery of a new genus. This finding highlights the lack of biodiversity data from understudied large rivers, particularly those affected by human and livestock activities. The data obtained from this study may contribute to the development of biological indices for water quality assessment in this country, supporting the maintenance of good water quality and benefiting the communities and ecosystems that depend on these water resources.

## Supplementary Material

ieag048_Supplementary_Data

## Data Availability

All data supporting the findings of this study are publicly available. DNA sequence data generated and analyzed during this study have been deposited in GenBank under accession numbers PX655555 and PX655556. These datasets can be accessed directly through the GenBank database. *Gattollius thailandicus* gen. nov. *et* sp. nov., SEMs of legs: A) foreleg, B) middle leg, C) hind leg, D) fore claw. *Gattollius thailandicus* gen. nov. *et* sp. nov., SEMs of fore femur: A) setae on the outer margin of the base, B) setae on the outer margin of the mid-section, C) setae on the dorsal margin of the apex, D) setae on the ventral margin of apex. *Gattollius thailandicus* gen. nov. *et* sp. nov., SEMs of femur: A) femoral villopore gills of foreleg, B) chloride cells near FVGs of foreleg, C) femoral villopore gills of middle leg, D) femoral villopore gills of hindleg. *Gattollius thailandicus* gen. nov. *et* sp. nov., SEMs of femoral villopore gills: A) femoral villopore gills of foreleg, B) femoral villopore gills (ventral view), C) femoral villopore gills (lateral view), D) setae on femoral villopore gills. *Gattollius thailandicus* gen. nov. *et* sp. nov., SEMs of legs: A) inner margin of fore tibia, B) setae on the tibia of foreleg, C) setae on the inner margin of fore tarsus, D) setae on the inner margin of tibia (apex) and tarsus of middle leg. *Gattollius thailandicus* gen. nov. *et* sp. nov., SEMs of accessory gills: A) prosternum of female larva, B) foreleg of female larva, C) prosternum of male larva, D) foreleg of male larva. *Gattollius thailandicus* gen. nov. *et* sp. nov., SEMs of abdomen and gills: A) abdomen segments (lateral view), B) gill I to II (lateral view), C) left gill I (dorsal view), D) right gill VII (dorsal view). *Gattollius thailandicus* gen. nov. *et* sp. nov., gills morphology: A) gill I, B) gill II, C) gill III, D) gill IV, E) gill V, F) gill VI, G) gill VII, H) tergum V, I) sternum V. *Gattollius thailandicus* gen. nov. *et* sp. nov., SEMs of sternum and paraproct: A) sternum IX (lateral view), B) paraproct and base of caudal filament, C) sternum X and paraproct (ventral view), D) paraproct (lateral view). Type locality of *Gattollius thailandicus* gen. nov. *et* sp. nov. *Gattollius thailandicus* gen. nov. *et* sp. nov., tracheation of accessory gills visible by methylene blue: A) overview of accessory gills, B) labium gills, C) coxal gills, D) femoral villopore gills. Habitat of *Gattollius thailandicus* gen. nov. *et* sp. nov.: A) type locality, B) sampling site, C) microhabitat, D) substrate.

## Nomenclature

This paper and the nomenclatural act(s) it contains have been registered in Zoobank (www.zoobank.org), the official register of the International Commission on Zoological Nomenclature. The LSID (Life Science Identifier) number of the publication is: urn:lsid:zoobank.org:pub:151B4A89-C7CD-48F9-9452-EFFB3D004E50.
